# Fatal Asymptomatic Myocardial Infarction Following Stroke in a Fontan Adolescent: A Case of Occult Blastomycosis Infection

**DOI:** 10.1155/crpe/5001168

**Published:** 2025-11-27

**Authors:** Hala K. El Mikati, Kyla Cordrey, Regi Ramanathan, Raja Rabah, Caren S. Goldberg, Nathaniel Sznycer-Taub

**Affiliations:** ^1^Department of Pediatrics, Congenital Heart Center, University of Michigan, Ann Arbor, Michigan, USA; ^2^Department of Pathology, Pediatric and Perinatal Pathology, University of Michigan, Ann Arbor, Michigan, USA

**Keywords:** cerebrovascular events, Fontan circulation, myocardial infarction, single ventricle physiology

## Abstract

Myocardial infarction from coronary ischemia in pediatric patients with Fontan circulation is a rare diagnosis with scarce data reported on it. We describe a case of a teenage male who initially presented with an acute occlusive right middle cerebral artery (MCA) stroke with a history of nonadherence to aspirin. He underwent a thrombectomy shortly after presentation and was not restarted on anticoagulation due to hemorrhagic transformation of the stroke postprocedure. He had no residual neurologic deficits. On the sixth day of his admission and without prior symptoms, he had a sudden cardiac arrest with pulseless electrical activity (PEA). After 80 min of resuscitation, including an unsuccessful attempt to cannulate for extracorporeal membrane oxygenation (ECMO), he was pronounced dead. On autopsy, he was noted to have an acute premortem right coronary artery (RCA) occlusion and blastomycosis infection of the lung. Patients with Fontan circulation are known to have a higher risk for thrombotic events, especially when not on any antiplatelet therapies, as well as immunologic derangements. This case suggests that an indolent infection in the setting of passive blood flow within the Fontan circulation may have contributed to a prothrombotic state which may have ultimately led to the stroke and myocardial infarction.

## 1. Background

Myocardial infarction from coronary ischemia in pediatric patients palliated with a Fontan procedure has rarely been reported. We describe a case of a teenage male who had an in-hospital cardiac arrest and was found to have right coronary artery (RCA) occlusion and myocardial infarction in the setting of a pulmonary blastomycosis infection on postmortem examination.

## 2. Case Presentation

### 2.1. Initial Presentation and Pediatric Intensive Care Unit (PICU) Course

We describe the case of a 14-year-old male with a history of hypoplastic left heart syndrome (HLHS) with mitral stenosis/aortic stenosis (MS/AS), who was initially palliated with a Norwood arch reconstruction and a right ventricular–pulmonary artery conduit (Sano shunt) at eight days of life. His course was complicated by the need for conduit revision at 2 weeks of age. He then underwent a hemi-Fontan at 7 months of age. He was noted to have restriction across his atrial septum, and he underwent an atrial septostomy at the age of 2 years, followed by a lateral tunnel fenestrated Fontan 6 months later. At the age of 5 years, he underwent fenestration closure with an Amplatzer Duct Occluder II due to hypoxia with improvement in his baseline saturations.

At the age of 14 years, he presented to the emergency department (ED) with new symptoms of left-sided arm and leg weakness. On examination, he had left-sided facial droop, loss of light touch on the left side, and right gaze deviation as he was unable to cross to midline. He reported that he had been experiencing intermittent headaches and neck pain for 2 months and had not been taking his prescribed aspirin. A CT/CTA was obtained in the ED which diagnosed a right middle cerebral artery (MCA) stroke. The patient was then transferred to a referral center for further management. Upon arrival, his labs showed a white blood cell count of 8.6 k/μL, with a low lymphocyte count of 17.4% that later trended down to 8.3% (normal 25%–45%), hemoglobin of 15.6 g/dL, and a platelet count of 171 k/μL. A coagulation panel was sent, and it showed antithrombin activity of 83%, protein C activity of 84%, free protein S antigen of 91%, and a negative phospholipid neutralization, all of which were within normal limits. C-reactive protein was mildly elevated at 2.2 (normal < 0.6 mg/dL). The comprehensive viral panel was negative for viruses. No blood cultures or other infectious studies were sent, given the lack of a history of fever and other signs of infection.

After presentation, he underwent emergent thrombectomy with interventional radiology with full reperfusion of the MCA; however, the procedure was complicated by MCA vasospasm requiring intra-arterial verapamil initiation. On follow-up imaging, he was noted to have hemorrhagic transformation of the stroke without midline shift. Anticoagulation and antiplatelet therapies were consequently held. Serial imaging showed new smaller bleeds around the hemorrhagic transformation. The left-sided weakness improved postprocedurally, without any additional neurological deficits. An echocardiogram obtained during the admission demonstrated a very small residual fenestration through his Fontan pathway with right-to-left shunting by bubble study (Supporting Information (available here)). Right (systemic) ventricular function was low-normal without any noted regional wall motion abnormalities on that echo, and antegrade flow was seen into both coronary arteries. He was transferred to the acute care cardiology floor 72 h after his initial presentation.

### 2.2. Acute Care Cardiology Unit (ACCU) Course

Based on interdisciplinary discussions with neurology and neurosurgery, antiplatelet and other anticoagulants were not prescribed, given the hemorrhagic transformation. He described daily headaches; however, his neurological exams showed no new deficits.

### 2.3. Code

In the early hours of the sixth day of his admission, he had a sudden cardiac arrest, without any prior cardiorespiratory symptoms, with pulseless electrical activity (PEA). Pediatric Advanced Life Support (PALS) was initiated with chest compressions and intravenous epinephrine. He was subsequently orally intubated. During the resuscitation, he was noted to have ventricular fibrillation, and multiple defibrillations were attempted, but he did not achieve return of spontaneous circulation (ROSC). The decision was made for cannulation onto venoarterial extracorporeal membrane oxygenation (VA-ECMO). Cannulation was ultimately unsuccessful due to femoral vessel occlusion. He was declared dead after 80 min of resuscitation. Afterwards, his parents requested an autopsy.

### 2.4. Postmortem Examination

**Figure 1:**
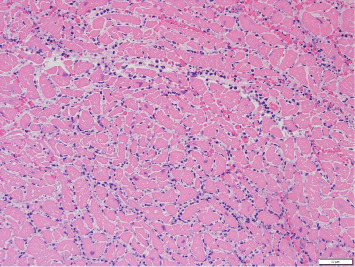
Section of the right ventricle with large areas of ischemic necrosis with neutrophilic infiltrate.

**Figure 2:**
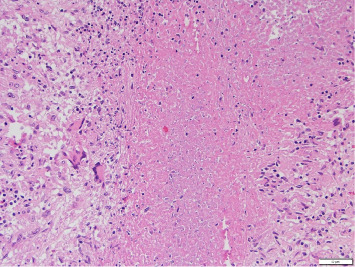
Section from the right upper lobe of the lung showing granulomatous and necrotizing inflammation with fungal organisms positive with Grocott–Gomori's methenamine silver (GMS) stain.

**Figure 3:**
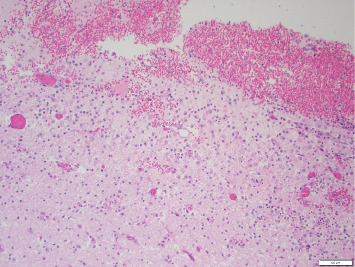
Brain section showing global hypoxic ischemic changes and a subacute hemorrhagic area in the right basal ganglia.

An occlusive thrombus was noted in the proximal segment of the RCA. The left coronary arteries were patent. Microscopically, the right ventricle showed large areas of ischemic necrosis with neutrophilic infiltrate consistent with a recent myocardial infarction around 24 h old (Figure 1). Fungal stains were negative, and no epithelioid or necrotizing granulomas were noted. Sections of the heart showed interstitial scarring and mild mononuclear inflammation with scattered giant cells suggestive of prior myocarditis. The findings suggest a possible injury related to prior fungal or giant cell myocarditis. A localized area of consolidation was noted in the right upper lobe of the lung, and microscopic examination revealed granulomatous and necrotizing inflammation with fungal organisms (broad-based budding yeast forms) positive with Grocott–Gomori's methenamine silver (GMS) (Figure 2). Morphologically, these structures were suggestive of blastomycosis. Brain examination showed recent and global hypoxic ischemic changes and a subacute hemorrhagic area in the right basal ganglia (6.6 cm) (Figure 3).

## 3. Discussion

This is an unusual case of a teenage male with Fontan palliation, admitted initially for an acute stroke, who ultimately experienced a PEA arrest leading to his death. This was thought to be likely from a coronary thrombus and consequent MI. Although he did not report any infectious symptoms, his postmortem autopsy indicated a chronic fungal pulmonary infection which may have contributed to a hypercoagulable state. Infections are a known trigger to the extrinsic coagulation cascade, suppression of anticoagulant pathways, fibrinolysis suppression, and cytokine-induced coagulation amplification [1, 2]. This presentation is worth sharing, given some atypical findings, including coronary ischemia in a young patient with Fontan circulation, possibly potentiated by a fungal infection and lack of adherence to antiplatelet therapy.

The patient's initial presentation with stroke is contextualized by potential aspirin nonadherence and opportunity for paradoxical embolism through a residual leak following Fontan fenestration closure. Previous studies show that antiplatelet therapy with aspirin decreases the risk for cerebrovascular events in children with single ventricles. Notably, due to his hemorrhagic transformation, the patient had remained off anticoagulation and antiplatelet therapies throughout admission, possibly increasing the risk of thrombosis and coronary artery occlusion. Although cerebrovascular events are known complications in the setting of aspirin nonadherence [3], myocardial infarcts are not as well reported in the literature in this population. Less is known or reported about embolic myocardial ischemic events in the single ventricle patient population. There are few case reports documenting myocardial infarcts in the single ventricle population [4–6]; however, in those that are published, few patients present without associated cardiac symptoms. The patient's cardiac arrest etiology was unexpected, given the notable absence of preceding chest pain or dyspnea; he only reported headaches and nausea, which he had experienced intermittently with his stroke recovery. In addition, the patient's initial cardiac arrest rhythm was marked bradycardia followed by PEA. Acute coronary ischemia has been associated with PEA in the setting of prearrest severe left ventricular dysfunction [7]; this patient, however, had only moderate systemic ventricular dysfunction on his admission echocardiogram. His coronary ischemia can also explain the conversion to V-fib during his arrest, although that was short-lived.

Lastly, results from his autopsy also noted blastomycosis lung disease and concerns for a nonacute myocarditis. We cannot determine whether his initial stroke was a septic thromboembolic event or whether an ongoing fungal infection predisposed him to clot development. It is noteworthy that he had a fungal infection and granulomatous changes in his lungs and signs of myocarditis which may have been consistent with an indolent fungal infection. Adult Fontan patients have been shown to have lymphopenia [8], and some studies suggest its presence in pediatric patients as well, even in the absence of protein-losing enteropathy (PLE). The mechanism of this lymphopenia is not clearly delineated in the literature though most often associated with PLE, plastic bronchitis, or portal hypertension. Some authors hypothesize that in the setting of an obligate venous congestion and hypertension in the Fontan circulation, tissue fluid clearance becomes impaired, leading to lymphatic disorders. Consequently, the lymphatic system is decompressed in the gut where there is less resistance, leading to lymphopenia. This, however, is more likely the mechanism of lymphopenia associated with overt PLE and may fall short when evaluating patients with lymphopenia and otherwise no signs of PLE. Others have hypothesized that chronic inflammation and splenic sequestration lead to lymphopenia [9, 10]. Despite these theories related to lymphopenia, there are no data demonstrating that patients with Fontan circulation are at an increased risk for fungal infection [8]. The relative immunocompromised state of Fontan patients deserves further investigation, and this case presentation raises the question of whether some indolent fungal infections can go undetected and may result in severe presentations, especially as it relates to thrombosis risk in an already prothrombotic low flow state. Our patient presented with lymphocytopenia but had otherwise no clinical signs of infection to guide our team to infectious screening. Therefore, in Fontan patients presenting with a significant thrombotic event, clinicians should consider screening for other causes for a hypercoagulable state including occult indolent infections, as treating an underlying infection could be a critical component of mitigating future thrombosis risk.

## Nomenclature

HLHS: Hypoplastic left heart syndrome
MS/AS: Mitral stenosis/aortic stenosis
ED: Emergency department
CT: Computed tomography
CTA: Computed tomography angiography
MCA: Middle cerebral artery
MI: Myocardial infarction
PICU: Pediatric intensive care unit
ECG: Electrocardiogram
GMS: Grocott's–Gomori's methenamine silver

## Supporting Information

Short clip of the bubble study showing bubbles in the RV.
